# Laser direct writing and Raman Stokes contrast screening of quantum emitter sites in hBN

**DOI:** 10.1186/s11671-026-04530-9

**Published:** 2026-03-26

**Authors:** Tadas Paulauskas, Julius Janušonis, Edgaras Markauskas, Viktorija Nargelienė, Vakaris Šilys, Ifra Bibi, Danielis Rutkauskas, Skirmantas Keršulis, Virginijus Bukauskas, Martynas Talaikis

**Affiliations:** Center for Physical Sciences and Technology, Sauletekio al 3, LT- 10257 Vilnius, Lithuania

**Keywords:** Laser direct writing, Single-photon emitters, Micro-Raman, hBN, Quantum technologies

## Abstract

Laser direct writing (LDW) enables the spatially defined creation of room-temperature single-photon emitters (SPEs) in hexagonal boron nitride (hBN). However, the rapid characterization of written sites remains a bottleneck, and the available toolset for efficient screening is limited. Here, we demonstrate a streamlined LDW workflow utilizing single-shot pulses combined with a confocal screening technique that exploits the hBN E_2*g*_​ Stokes line to rapidly localize and map laser-modified regions without relying a priori on defect photoluminescence (PL). This approach enables the direct correlation of site morphology with PL hotspots, revealing that the emergence of single-photon emitters coincides with a threshold regime of minimal lattice modification. Micro-Raman spectral mapping further uncovers localized compressive strain surrounding these emission sites. We classify the generated defects into two families: narrowband “red” emitters (650–750 nm) with weak phonon sidebands (PSB), and 600–650 nm emitters with stronger vibronic coupling, both exhibiting linear polarization and high single-photon purity. These results establish a practical protocol for rapid prototyping, offering a valuable addition to the characterization toolkit for scalable quantum nanophotonics.

**Supplementary Information:**

The online version contains supplementary material available at 10.1186/s11671-026-04530-9.

## Introduction

Femtosecond laser direct writing (LDW) has emerged as a versatile technique for the maskless, scalable fabrication of nanophotonic devices and functional quantum structures [1, 2]. By confining energy deposition to laser focal or even sub-diffraction volumes, LDW allows for the precise modification of wide-bandgap semiconductors without the need for foreign-species implantation or complex lithography. Among solid-state hosts, hexagonal boron nitride (hBN) is of particular interest for LDW-based quantum prototyping, as it hosts room-temperature single-photon sources (SPS) with brightness and stability rivaling the nitrogen-vacancy (NV) center in diamond [3–6]. When integrated via LDW, these emitters offer a pathway toward compact components for quantum key distribution (QKD) and quantum random number generation (QRNG) [7, 8].

However, a common bottleneck in the LDW workflow is the rapid characterization of successfully written sites. While recent studies have demonstrated high-yield defect creation using femtosecond pulses, the process remains inherently stochastic near the optical breakdown threshold [9–12]. Conventional characterization relies on time-consuming photoluminescence (PL) mapping to locate active emitters within large arrays, often followed by cumbersome correlation of PL hotspots with sample morphology, thus hindering the rapid prototyping cycle required for device integration. Furthermore, while the structural origins of known hBN defects, such as the boron vacancy (V_B_^−^​) or carbon dimer (C_B_​C_N_​), are established, the microscopic nature of the technologically attractive narrowband “red” emitters (650–750 nm) generated by LDW remains debated [17–21]. Understanding the interplay between laser-induced lattice strain, damage thresholds, and spectral purity is essential for maturing LDW into a reliable manufacturing tool.

Here, we demonstrate a practical LDW and characterization workflow that addresses these challenges. We introduce an optical screening technique that exploits the intrinsic hBN E_2*g*_​ Raman Stokes line to rapidly flag laser-modified regions without relying on defect fluorescence a priori [22–25]. By operating in the near-threshold regime, we isolate high-purity single-photon emitters where the lattice modifications are minimal yet discernible via Stokes-based screening. Spectroscopic analysis classifies these LDW-generated defects into two groups: a class of narrowband red emitters (650–750 nm) with weak phonon sidebands (PSB), and a secondary family (600–650 nm) with stronger PSB. Polarization-resolved measurements confirm linear dipole emission for both families and show co-existing unpolarized broad background in some LDW-modified sites. Furthermore, micro-Raman spectral mapping reveals localized compressive strain, which we find correlates with reduced emitter formation probability [26, 27]. These results define a robust approach for the rapid prototyping and qualification of LDW-based quantum emitters for next-generation hBN nanophotonics.

## Materials and methods

hBN flakes (HQ Graphene) were mechanically exfoliated onto 0.5 mm fused-silica substrates. Substrates were cleaned in acetone/isopropanol/DI water and oxygen-plasma treated prior to exfoliation. After exfoliation, samples were baked in air at 500 °C for 10 min to reduce tape residues. Laser writing was performed with a 1030 nm femtosecond source (Pharos, Light Conversion, 250 fs, 602 kHz) operated in a single-shot mode using an internal pulse picker. The linearly polarized beam was attenuated to the desired pulse energy and focused through a 50×, NA 0.65 objective to a ~ 1.6 μm spot. Sites were written under ambient conditions with 5–7 μm spacing using motorized translation stages. Following writing, samples were annealed in flowing Ar (~ 200 mL/min): 20 °C min⁻¹ ramp to 1000 °C, 90 min dwell, then cooled to room temperature. Surface morphology was characterized by tapping-mode AFM using silicon cantilevers. Confocal fluorescence imaging and spectroscopy were performed on a custom inverted microscope using 532 nm CW excitation (typically 1 mW at the sample). Samples were scanned with a piezo stage (0.18 μm pixels, 1 ms dwell) and a 75 μm pinhole. Emission was collected through a long-pass filter (HQ545LP, Chroma) and detected using a single-photon avalanche photodiode (Tau-SPAD-50) for imaging/count-rate measurements. Spectra were acquired using a grating spectrometer with an EMCCD (Shamrock SR-303i (50 gr/mm) + Andor DU-897E-CS0-UVB), averaged over 10 acquisitions with 1 s integration each. Polarization-resolved spectra were recorded by rotating a linear polarizer in the detection path in 10° steps and averaging 10 spectra per angle (0.1 s each). E_2*g*_​ -based screening imaging, second-order correlation, and fluorescence lifetime measurements were performed on a separate custom confocal microscope (50 μm pinhole, NA 0.90 air objective). $\:{g}^{\left(2\right)}\left(\tau\:\right)$ measurements were carried out under CW excitation (520 nm, Integrated Optics) using a Hanbury Brown–Twiss (HBT) configuration with two single-photon detectors (Excelitas SPCM-AQRH-14). Timing was recorded with a time-correlated single-photon counting module PicoHarp 300. Radiative lifetimes were measured using pulsed 520 nm excitation (Picoquant LDH-D-C-520) and TCSPC detection. Details of $\:{g}^{\left(2\right)}\left(\tau\:\right)$ offline post-processing and fitting are provided in the Supplementary Information. Raman spectroscopy and mapping were performed using 532 nm excitation with a 2400 lines/mm grating and a high-NA objective using Renishaw inVia. Raman maps were acquired with 0.5 × 0.5 μm step size and 6 s integration per point at ~ 3 mW incident power. Wavenumbers were calibrated using the Si line at 520.7 cm⁻¹. Baseline correction and peak/intensity mapping processing are described in the SI.

## Fabrication and Raman-based screening of LDW sites

### Morphological evolution and surface topography

**Fig. 1:**
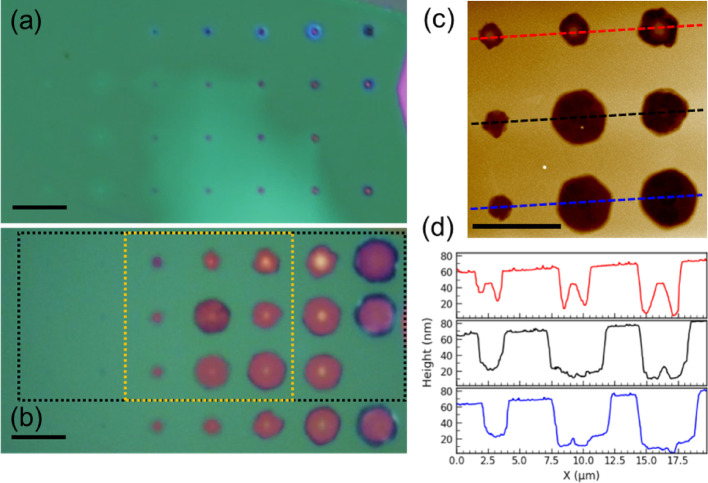
**a** Optical micrograph of LDW hBN before annealing. Seven spot columns are visible with pulse energy decreasing right to left. **b** Same area after annealing; dashed boxes mark regions scanned in later figures. Scale bars 7 μm. **c** AFM topography of the yellow central box in (**b**). **d** Line profiles across spots in (**c**) (matching colors). Note, the vertical axis shows relative surface height, not absolute flake thickness

All samples were processed using single-shot 1030 nm femtosecond LDW pulses. Pulse energies spanned 90–10 nJ, with finer increments employed to refine the near-threshold regime (< 50 nJ) where single emitters first appear. Optical micrographs acquired pre- and post-annealing (Fig. 1) illustrate the morphological evolution. In the representative hBN flake, seven columns (89.5 to 35.6 nJ) remain discernible prior to annealing (Fig. 1a). Post-anneal (Fig. 1b), higher-energy LDW sites enlarge markedly, whereas the lowest-energy columns show negligible change.

AFM topography of the central region (Fig. 1c–d) characterizes these features as shallow craters (~ 20–40 nm deep) whose lateral dimensions scale with pulse energy (~ 2–5 μm). At the lowest energies, only few-nanometer surface steps are observed, which often vanish upon annealing. Since the flake thickness (~ 160 nm) exceeds the crater depth, the modified regions remain well-confined above the substrate. Under identical experimental conditions, certain craters exhibit elevated central hBN topographies post-annealing, likely reflecting stochastic variations in the local material response. Notably, no rim pile-up or debris is evident, and some larger craters exhibit hexagonal outlines. Similar trends were observed across hBN flakes 50–500 nm thick. We hypothesize that laser-induced disorder and high local curvature accelerate hBN sublimation during the high-temperature anneal, potentially via volatile oxide formation, resulting in crater enlargement without material redeposition.

### Stokes-contrast mapping of LDW sites

**Fig. 2:**
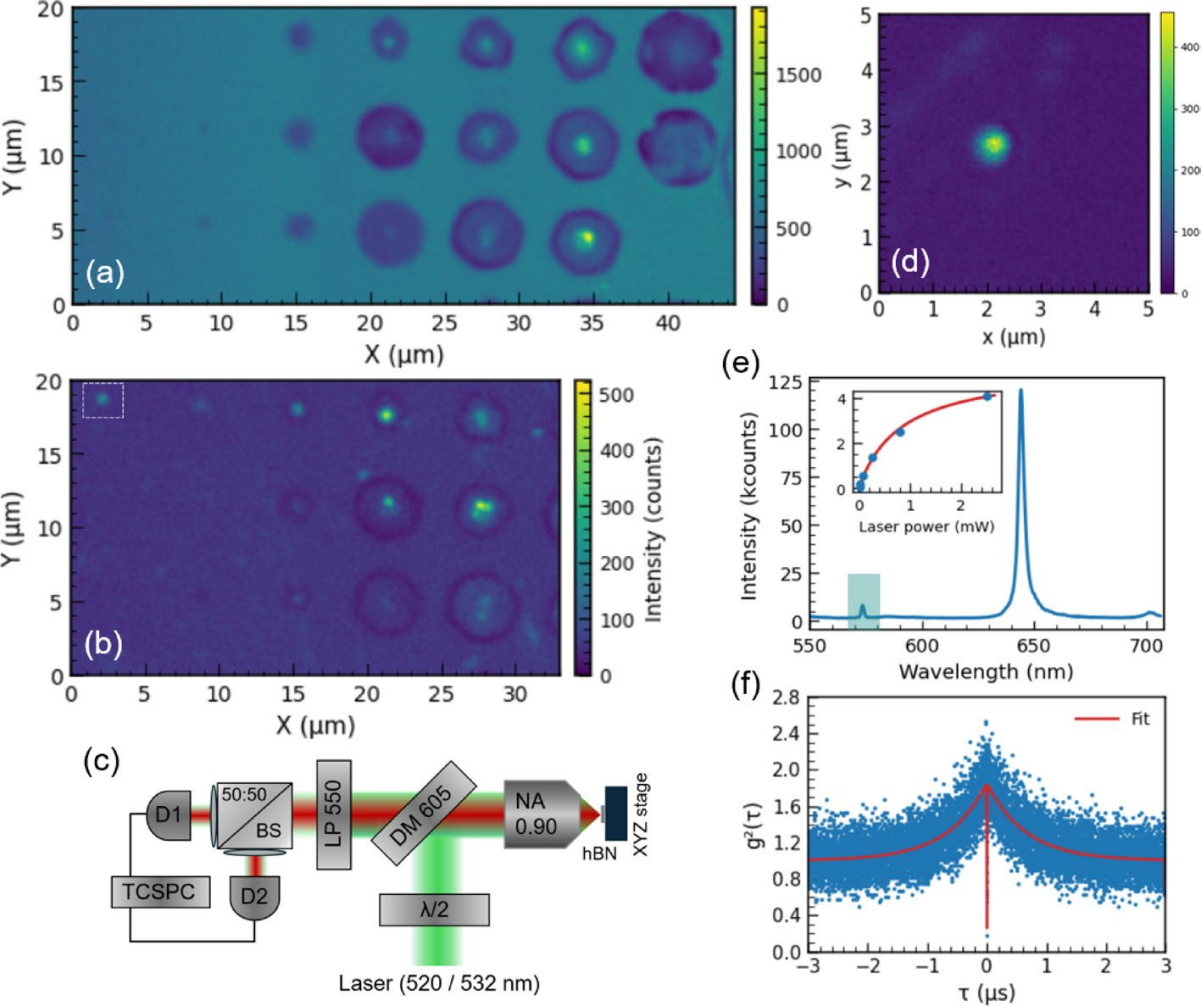
**a** Scanning confocal image of the region marked in Fig. 1b. **b** Same field of view (x = 0–30 μm) with the hBN Stokes contribution suppressed by rotating the half-wave plate. **c** Simplified optical layout for imaging and $\:{g}^{\left(2\right)}\left(\tau\:\right)$ measurements. **d** Confocal image of a single emitter indicated in (**b**). **e** Fluorescence spectrum of the defect in (**d**) with ZPL at 645 nm and PSB at ~ 700 nm; inset shows laser power dependence, vertical axis in 10^5^ cts/s. The hBN E₂_*g*_ Stokes peak is marked blue, used for contrast in (**a**). **f** Second-order correlation $\:{g}^{\left(2\right)}\left(\tau\:\right)$ of the defect with 3-level fit

Most LDW-modified regions in hBN, and indeed other morphological features such as grain boundaries or thickness variations, typically exhibit weak or negligible PL signal. Consequently, sub-bandgap excitation offers little to no contrast. Therefore, correlating defect PL with material morphology usually requires aligning pre-acquired images (e.g., optical or SEM) with subsequent scanning PL maps. Here, we introduce a practical confocal method to locate and map LDW-modified regions without relying on a priori defect PL. A key strength of this method is its compatibility with standard confocal scanning PL microscopes, requiring minimal optical layout modifications. The contrast exploits the hBN E_2*g​*_ Raman Stokes line (~ 560 nm for 520 nm excitation, ~ 573 nm for 532 nm), which is strongly co-polarized with the laser pump due to the in-plane isotropic hBN Raman tensor, alongside a slight leakage of *p*-polarized light through the long-pass dichroic mirror [22–25]. By setting the excitation to *p*-polarization with a half-wave plate, the E_2*g​*_ signal transmits through the dichroic while back-reflected laser light is blocked by a 550 nm long-pass filter. A simplified minimal optical layout for this E_2*g*_​-based screening is shown in Fig. 2c.

This E_2*g*_​-based mapping yields a bright bulk background with darker laser-written sites (Fig. 2a), primarily because the Raman signal scales with flake thickness. Contrast is further enhanced by crater-induced depolarization as well as reduced Raman excitation and collection efficiency in deformed regions. Additionally, thickness-dependent oscillations arising from pump standing waves and Stokes out-coupling effects may modulate the signal [24]. Defect PL above the dichroic cutoff (605 nm) may still appear in some spots if no short-pass filters are employed. This defect-based PL is visible as central hotspots in certain Fig. 2a craters, effectively dominating the local signal. Switching the excitation to *s*-polarization suppresses E_2*g*_ ​ transmission, eliminating the bright background and revealing the defect PL directly (Fig. 2b). If control over excitation polarization is required for defect PL analysis without the E_2*g*_​ signal, an additional long-pass filter (e.g., at 575 nm) can simply be inserted into the PL collection path.

As shown in Fig. 2a, sites created with pulse energies down to 35.6 nJ are readily identifiable via this method. Notably, below this energy threshold in our datasets we did not observe neither Stokes-contrast nor defect PL. This correlation remained consistent across various investigated hBN flakes, suggesting that lattice modifications required to host quantum emitters are linked to the structural changes detectable via Stokes-screening. Nevertheless, the method is intended as a rapid morphology mapping and localization tool rather than a standalone SPE selector. Since LDW sites may host single SPEs, multi-emitter ensembles, or no detectable PL, Raman screening is a practical tool for rapid targeting but not a substitute for traditional quantum optical verification.

A site hosting single emitter in this field of view is marked in Fig. 2b and imaged at higher magnification in Fig. 2d. Its spectrum (Fig. 2e) has zero-phonon line (ZPL) at 645 nm with a weak PSB near ~ 700 nm (“red” class of emitters). The E₂_*g*_ peak, marked in Fig. 2e, underpins the contrast formation in Fig. 2a. Power dependence of the SPE follows the standard saturation model, $\:I\left(P\right)={I}_{sat}P/(P+{P}_{sat})$, giving $\:{P}_{sat}\approx\:0.85$ mW and $\:{I}_{sat}$ ≈ 2.8 × 10^5^ cts/s, without polarization optimization and collection efficiency correction. The second-order correlation function, $\:{g}^{\left(2\right)}\left(\tau\:\right)$, was measured using 100 µW laser power at the objective (Fig. 2f) and at zero-delay it dips well below 0.5, indicating high single-photon purity. The noticeable photon bunching behavior at intermediate times is consistent with a minimal three-level system and was fitted with $\:{g}^{\left(2\right)}\left(\tau\:\right)=1-\left(1+a\right){e}^{-{\lambda\:}_{1}\tau\:}+a{e}^{-{\lambda\:}_{2}\tau\:}$ (see SI for additional data and analysis).

### Raman spectral imaging and local lattice strain

**Fig. 3:**
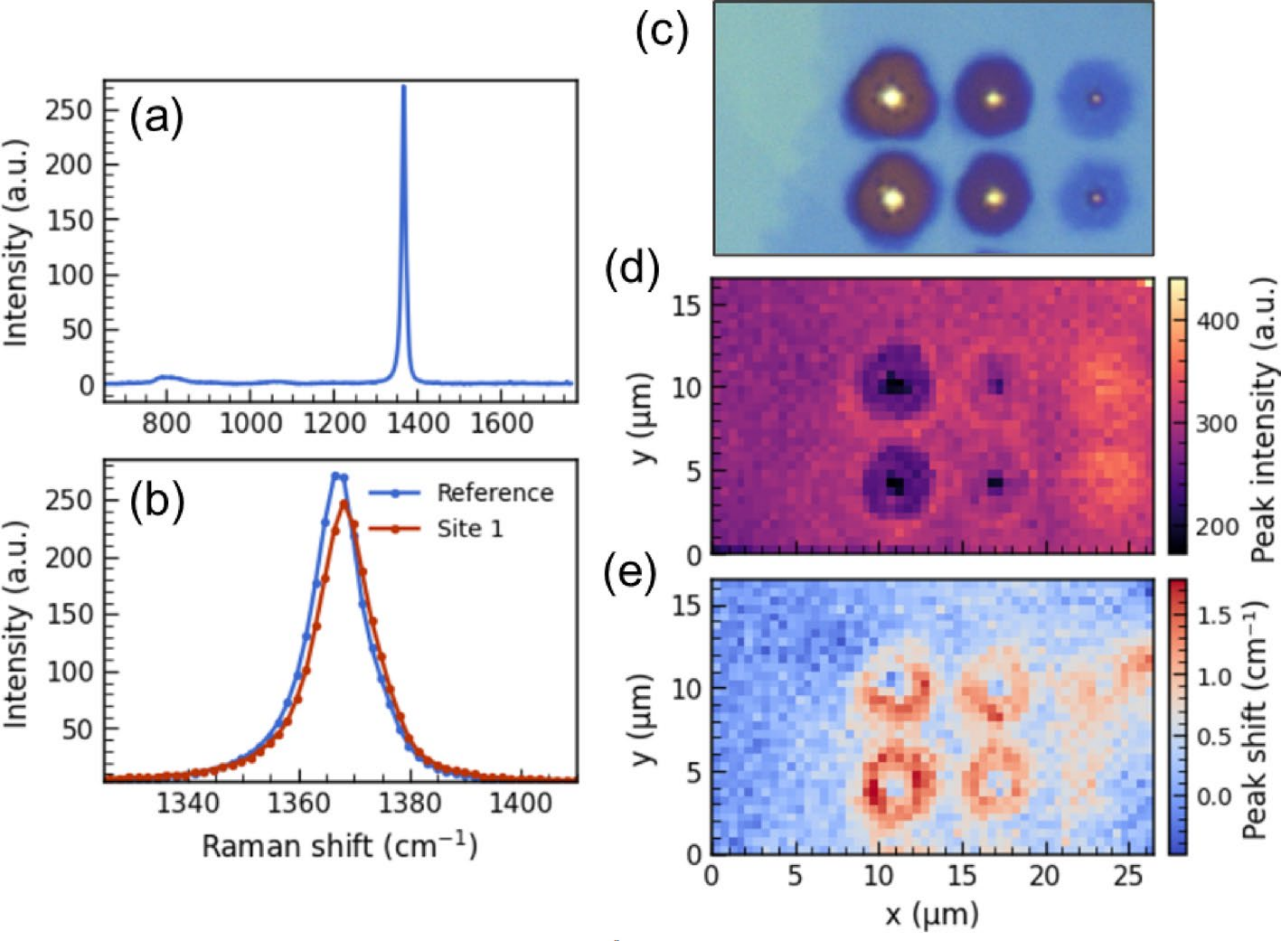
**a** Representative Raman spectrum of a laser-written site. **b** Overlay of a reference (unaffected bulk) and a laser-written spot, showing the typical E_2*g*_ peak redshift. **c** Optical micrograph of the area mapped in (**d**, **e**). **d** Raman map of E_2g_ peak intensity, highlighting variations near laser-written sites. **e** Raman map of E_2*g*_ peak position shift, revealing spatially varying redshifts. Reference E_2*g*_ peak position for this map is the unaffected region on the left from LDW sites

Micro-Raman spectral imaging was performed on the LDW-modified regions to further quantify lattice modifications (Fig. 3). The E_2*g*_ phonon mode showed spatially varying shifts and intensity changes, albeit no signatures of a secondary hBN phase (e.g., cubic-BN) or systematic defect-induced side bands were detected. A representative spectrum from a written site is shown in Fig. 3a. Figure 3b illustrates a typical E_2*g*_​ peak shift at written spots relative to an unaffected reference (~ 1367 cm⁻¹). Figure 3c shows the mapped area, with E_2*g*_​ intensity and peak-position maps in Fig. 3d–e derived from background-removed, peak-fitted spectra (see SI).

The E_2*g*_​ intensity generally dips at crater centers but can rise on rims and at the lowest-energy sites. Variations for the lowest-energy sites may point to the nonlinear thickness-dependent Raman response [24]. Notably, we observe localized positive E_2*g*_​ shifts (up to + 2 cm⁻¹) near larger crater bottoms. Interpreting the E₂_*g*_ shift, ($\:\varDelta\:\omega\:$), as a strain gauge, we estimate a compressive strain of ~ 0.1%, assuming a biaxial strain model ($\:{\upepsilon\:}=-\varDelta\:\omega\:/2\gamma\:{\omega\:}_{0}$) and literature Grüneisen coefficient $\:\gamma\:=1.04$ for multilayer hBN [26]. Under uniaxial strain the conversion factor is smaller, but the sign still indicates compression. We emphasize that the measured values are depth-averaged over the confocal volume of both pump and Stokes light, potentially underestimating the peak strain in localized LDW-affected layers.

These strain patterns indicate coexisting stress fields in laser-modified hBN. Interestingly, we observe that high-intensity defect PL hotspots typically occur within the central regions of LDW sites, rather than in the strained peripheral zones (Fig. 3b). This spatial distribution suggests that the likelihood of emitter formation during annealing is reduced at compressively strained sites. Since strain strongly modulates defect formation energies, the local compressive field may favor simple vacancies while increasing the formation energy barriers for the interstitial or complex substitutional defects required for single-photon emission in the visible spectral region.

## Spectroscopic characterization

### Emitter classification and spectral inhomogeneity

**Fig. 4:**
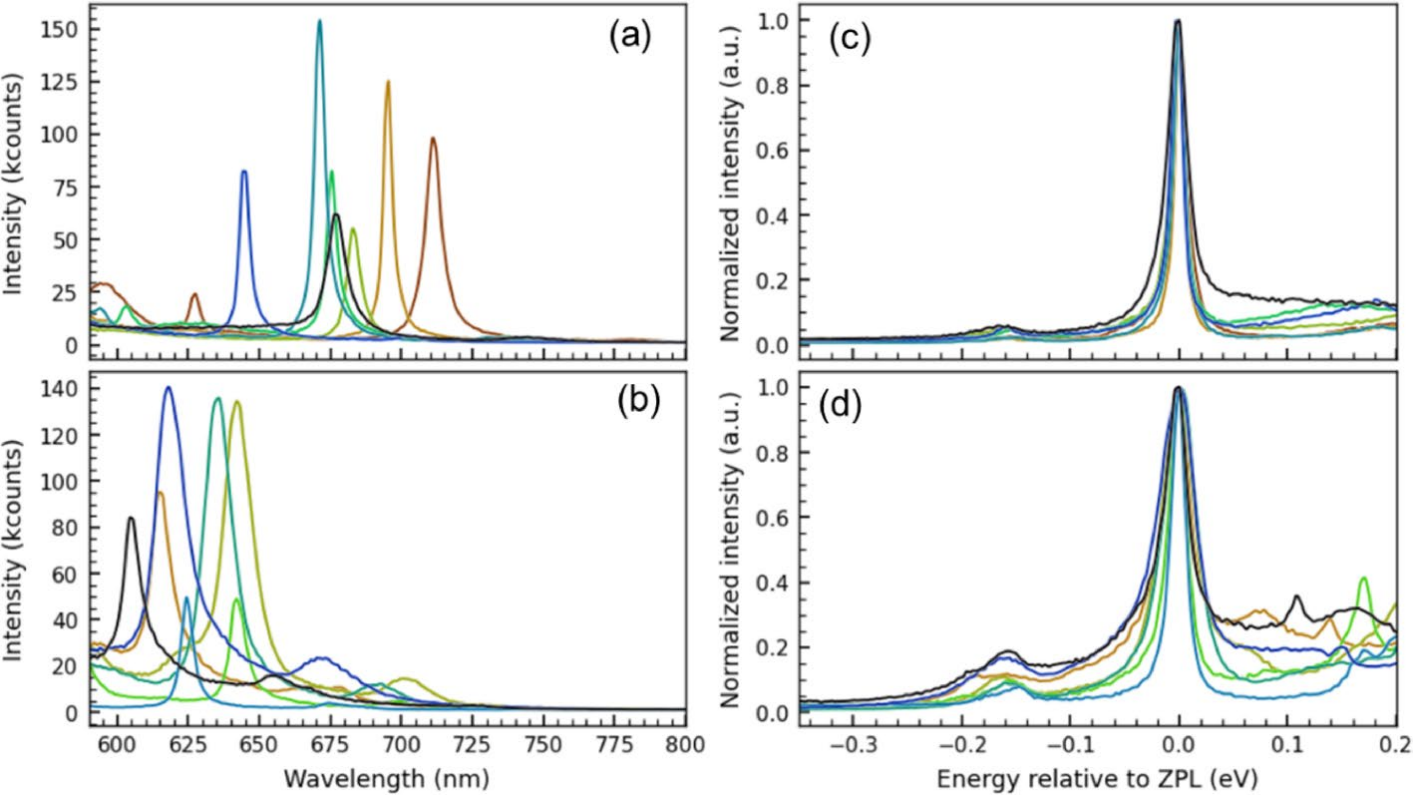
PL spectra of individual emitters at laser-written sites, grouped by phonon-sideband (PSB) strength. **a** Weak PSB. **b** Strong PSB. **c **and **d** Same spectra as (**a **and** b**), normalized and plotted on an energy axis with each trace aligned to its ZPL. Panels (**a **and** b**) and panels (**c **and** d**) share the same x-axis scale. Measurements were done at room temperature

We next analyze PL from LDW–fabricated single emitters. Figure 4 gathers spectra of two spectral families distinguished from typical “green” emitters by their markedly more pronounced ZPL and suppressed PSB. We further classify these families by ZPL position and PSB strength: (i) red emitters at ~ 650–750 nm with a clearly weak PSB (Fig. 4a), and (ii) emitters at ~ 600–650 nm with a stronger PSB (Fig. 4b). The excitation polarization was fixed for all measurements, thus, relative intensities may reflect excitation-coupling biases for differently oriented dipoles. To compare vibronic structures, we normalize each spectrum and plot them on an energy axis aligned to the ZPL (Fig. 4c–d). Both families exhibit PSB features ~ 170–180 meV from the ZPL, consistent with coupling to high-DOS phonons of bulk hBN [20, 28]. Regarding microscopic origin, DFT studies have proposed carbon-related centers (e.g., carbon trimers or donor–acceptor pairs) as candidates for weak-PSB red emitters, which is broadly consistent with our spectral data [18, 19].

Despite these shared characteristics, we observe a significant spread in ZPL positions and linewidths (FWHM) across the dataset. This spectral diversity is not unique to the LDW technique but is a pervasive feature of emitters in hBN, routinely reported across various fabrication methodologies including electron irradiation, ion implantation, and thermal annealing [5, 6, 9–12]. This universality suggests that the variations stem from local environmental heterogeneity, such as strain fields and charge fluctuations from nearby defects, as well as the potential existence of multiple stable atomic configurations (e.g., distinct defect-impurity complexes or defect pairs with varying separations) that possess slightly distinct transition energies [28, 29].

### Excited state lifetimes

**Fig. 5:**
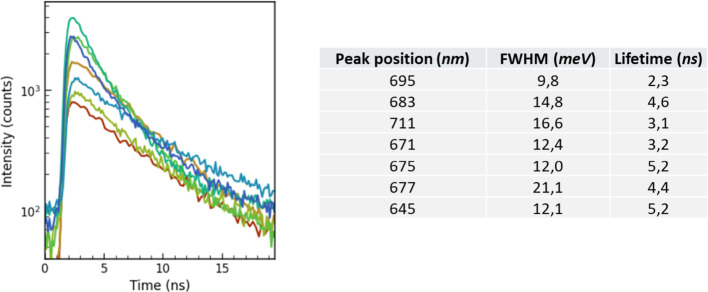
Time-resolved PL intensity of emitters with weak PSB, obtained at room temperature. Decay traces are fitted with monoexponential (listed in Table 1)

Excited-state lifetimes for the red emitters were quantified using pulsed 520 nm laser excitation (Fig. 5). The decay traces are well-described by a single-exponential model, yielding lifetimes in the ~ 2–5 ns range (Table 1). The single-exponential behavior indicates a dominant radiative decay channel characteristic of high-purity quantum emitters. For completeness, Table 1 also lists ZPL positions and Lorentzian ZPL FWHM. We observe no systematic correlation between lifetime, ZPL position, and FWHM. The variation is likely attributable to differences in the local density of optical states (LDOS) arising from the emitters’ varying depths within hBN or proximity to the dielectric interface, and not necessarily intrinsic to the defect structure itself [30].

### Spectrally resolved PL polarization

**Fig. 6:**
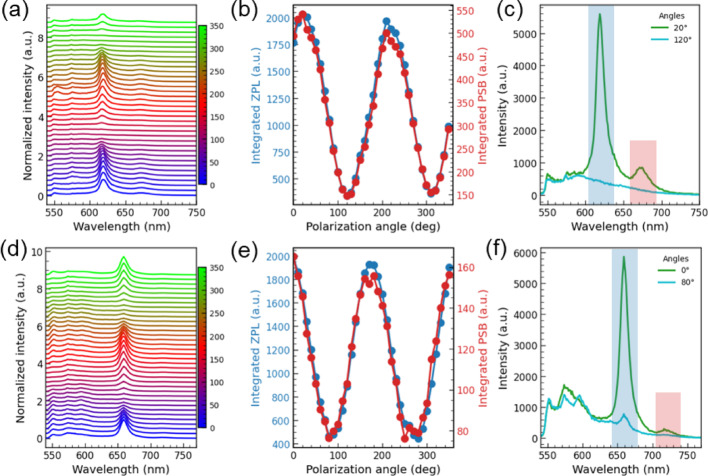
Spectrally resolved PL polarization for emitters with strong (**a**–**c**) and weak (**d**–**f**) PSB. **a**, **d** Polarization-dependent spectra (linear polarizer stepped by 10°), globally normalized. **b**, **e** ZPL and PSB intensities vs. polarization angle, integrated over the bands marked in (**c**, **f**); background was not fitted/subtracted. **c**, **f** Spectra at angles corresponding to each emitter’s maximum and minimum ZPL intensity

Spectrally resolved PL polarization measurements were performed on both emitter families. Representative datasets for strong-PSB (Fig. 6a–c) and weak-PSB (Fig. 6d–f) cases were acquired by stepping a linear polarizer in 10° increments. Both emitters exhibit clear linear-dipole behavior: the angle dependence of the ZPL-integrated intensity (background not subtracted; integration bands marked in Fig. 6c, f) shows pronounced maxima/minima, and the PSB-integrated intensity follows the same angle without a visible offset. This alignment implies that the dominant phonon-assisted transitions inherit the ZPL dipole orientation, suggesting that electron–phonon coupling does not strongly rotate the optical selection rules for these modes. Note, however, that 10° steps can miss the true extrema, so maxima/minima need not be separated by exactly 90°.

The degree of linear polarization (DoLP) for hBN emitters seldom reaches unity, as noted in the literature [5, 6, 31–33]. Several mechanisms can contribute to incomplete DoLP: (i) intrinsic PL depolarization processes, (ii) an unpolarized background that sets a floor at the polarization minimum, or (iii) experimental geometry effects, such as the projection of an out-of-plane dipole component. Our spectrally resolved measurements identify broad, ZPL-free emission bands peaking in the 580–600 nm window as the primary unpolarized background for the emitters in Fig. 6c, f. This background is detrimental to QKD protocols because it degrades single-photon purity, possibly explaining why $\:{g}^{\left(2\right)}\left(0\right)$ values saturate near ~ 0.2 for some yellow–red hBN emitters [5, 6]. Consequently, identifying and suppressing these broad emission defects is important for future fabrication optimization, or suggests a shift to NIR emitters and using longer-wavelength excitation to avoid this spectral window. Additionally, the incomplete suppression of ZPL and PSB features at polarization minima (Fig. 6f) points to a combination of intrinsic depolarization and residual background (mechanisms i and ii) limiting the contrast.

### Multiple-emitter sites and spectral statistics

**Fig. 7:**
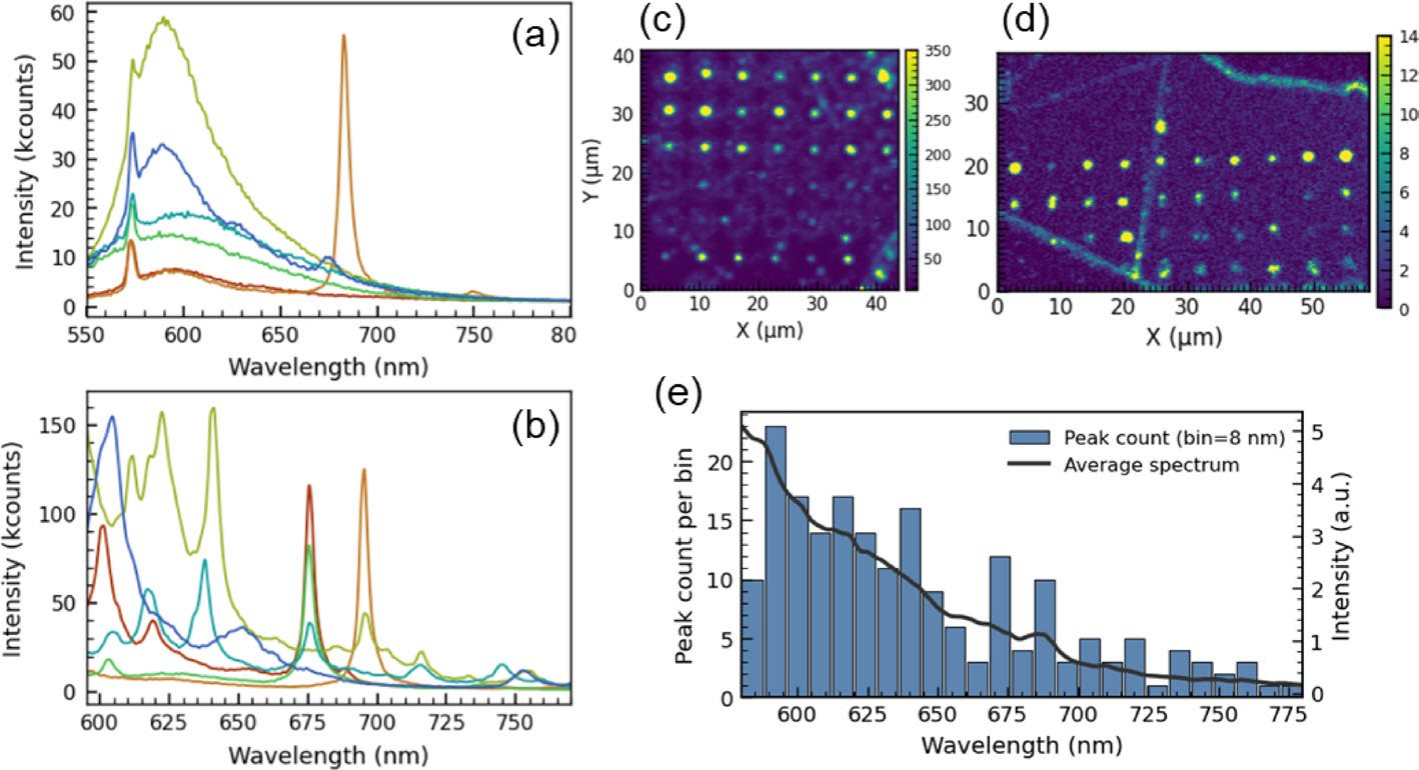
**a** Representative broad emissions peaking at ~ 580–600 nm from LDW sites; sharp feature at ~ 575 nm is E₂_*g*_ Stokes line. **b** Spectra illustrating multiple emitters within a single site and repeatedly observed red emitters with nearly identical ZPL positions. **c **and** d** Confocal PL images of written flakes showing bright emission hotspots. **e** ZPL histogram (8 nm bins, 580–800 nm range) and the average spectrum (solid line)

This section summarizes the distribution of emission signatures observed across the written sites, including SPE-like spectra, multi-emitter spectra that are more common at higher pulse energies, and broad, ZPL-free emission. The latter appears frequently peaking at ~ 580–600 nm (Fig. 7a), shows no polarization dependence (cf. Figure 6), and can persist as a broad background for polarized emitters, as discussed above. Exciting such sites with a 637 nm laser does not reproduce them next to the E₂_*g*_ Stokes line (at ~ 700 nm), ruling them out as, e.g., defect-based Raman modes. A plausible origin is PL from a defect center, or a family of defects, with a large Huang–Rhys factor and strong electron–phonon coupling. Vacancy-based emitters tend to have a large Huang–Rhys factor, one such well known example is $\:{V}_{\mathrm{B}}^{-}$ in hBN, which exhibits a broad ZPL-free band near 800 nm [13–15].

At higher laser pulse energies, spectra frequently display multiple ZPLs within a single site (Fig. 7b), typically unresolved spatially in confocal scans (examples in Fig. 7c–d). Interestingly, some multiple-emitter sites show repeated exact ZPL energies (up to our spectral resolution), e.g., at ~ 675 and ~ 695 nm (Fig. 7b). Given the general ZPL spread discussed earlier, such recurrence may suggest identical microscopic centers under very similar local strain. We note that in our experimental study we did not employ energies that create exceedingly large craters in hBN, as some previous studies have done. In those higher-energy cases, the craters show pronounced material pileups and folded layers at crater edges, which tend to host a high density of defects and produce an almost continuous bright PL rim. Here, in contrast, for the multiple-emitter sites at larger energies the emission originates mostly from the central spot. This can be seen in Fig. 7c–d as larger hot-spot emission. Future studies will aim to carry out ODMR on LDW-created SPEs and such multiple-emitter hot spots, which may be interesting for magnetic sensing applications and to help deduce their atomic origin.

Figure 7e compiles ZPL statistics across flakes, incorporating both single and multiple-emitter sites, and provides a quantitative overview of the LDW-generated spectral landscape in our experimental regime. Emitters occur most frequently in the 580–650 nm range, where the average spectrum correspondingly exhibits the highest PL intensity. A secondary clustering is observed in the red band (650–725 nm), accompanied by a distinct spectral feature in the average intensity around 675–700 nm. Considering the typical spread of ZPLs in hBN, this secondary clustering likely indicates a specific “red” defect structure, or a family of defects with similar transition energies, that is preferentially generated by the LDW and annealing process.

## Conclusions

In conclusion, we have demonstrated a workflow for femtosecond laser direct writing (LDW) of quantum emitters in hBN that facilitates the rapid post-process identification of written sites. Using an optical screening modality based on the intrinsic hBN E_2*g*_​ Raman Stokes line, we map laser-modified regions without relying a priori on defect photoluminescence, adding a simple and broadly implementable tool to the LDW characterization toolkit. This approach reveals that the emergence of single-photon emitters coincides with a threshold regime of minimal lattice modification, whereas higher pulse energies more frequently yield multi-emitter spectra and broadband backgrounds. The generated emitters fall into two families: narrowband red emitters (650–750 nm) with weak phonon sidebands and a 600–650 nm family with stronger vibronic coupling, both exhibiting linear polarization and high single-photon purity. However, a broadband unpolarized background centered at 580–600 nm can persist in some sites, degrading polarization contrast. Micro-Raman mapping reveals localized E_2*g*_​​ shifts consistent with compressive strain, yet these depth-averaged fields are insufficient to explain the ZPL spectral diversity, pointing to atomic-scale environmental heterogeneity as the dominant factor. Together, these results establish LDW combined with Raman-based screening as a practical characterization approach for qualifying hBN quantum emitter sites, paving the way for further advancements on LDW-based integrated nanophotonic devices.

## Supplementary Information

Below is the link to the electronic supplementary material.

Supplementary Material 1

## Data Availability

Data will be available on request.
