# Supplementary material for: Laser direct writing and Raman Stokes contrast screening of quantum emitter sites in hBN
Source: Discov Nano. 2026 Mar 26;21(1):85. doi: 10.1186/s11671-026-04530-9 (PMC13022109; doi:10.1186/s11671-026-04530-9)
Supplement: Supplementary file 1 — Supplementary Material 1 [file 11671_2026_4530_MOESM1_ESM.docx]

**Supplementary Material**

Laser Direct Writing and Raman Stokes Contrast Screening of Quantum Emitter Sites in hBN

Tadas Paulauskas,* Julius Janušonis, Edgaras Markauskas, Viktorija Nargelienė, Vakaris Šilys, Ifra Bibi, Danielis Rutkauskas, Skirmantas Keršulis, Virginijus Bukauskas, Martynas Talaikis

Center for Physical Sciences and Technology, Sauletekio al 3, Vilnius LT-10257, Lithuania

*Corresponding author: [tadas.paulauskas@ftmc.lt](mailto:tadas.paulauskas@ftmc.lt)

**Contents**

1. **Second-order correlation measurements**
2. **Raman data**
3. **Polarization-resolved photoluminescence**
4. **Second-order correlation measurements**

Second-order correlation function, $g^{\left( 2 \right)}\left( t \right)$, measurements were performed using a standard Hanbury Brown and Twiss (HBT) configuration. The emission was partitioned using a free-space 50:50 beam-splitter and detected by two single-photon counting modules (SPCM-AQRH-14, Excelitas), connected to a PicoHarp 300 (PicoQuant) time correlated single-photon counting module.

Raw time-tagged data (.ptu files) were extracted and processed using the readPTU library [1]. The experimental $g^{\left( 2 \right)}\left( t \right)$ data shows photon antibunching at short timescales and bunching at intermediate timescales (Fig. S2). This behavior was modeled using a standard 3-level system (Fig. S1), where the second-order correlation function is described by [2,3]:

$g^{\left( 2 \right)}\left( t \right)=\frac{p_{2}\left( t \right)}{p_{2}\left( \infty\right)}=1-\left( 1+a \right)e^{-\lambda_{1}t}+ae^{-\lambda_{2}t}$. (S1)


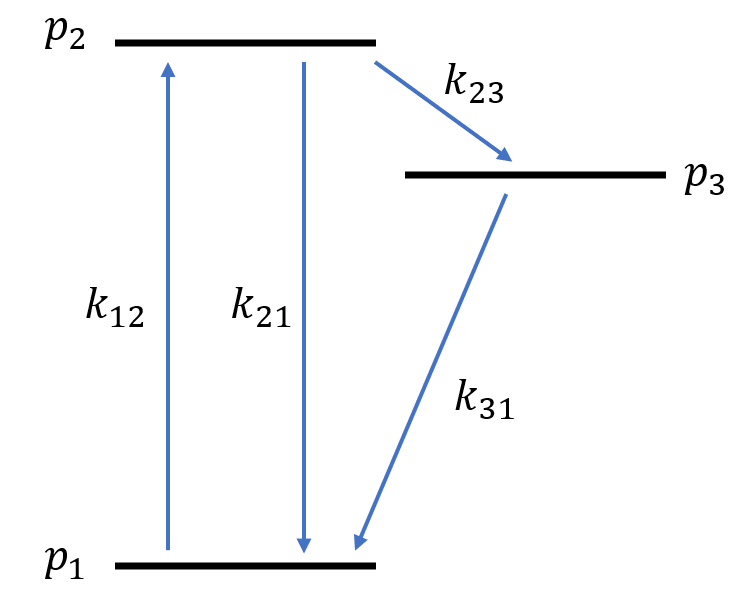


Fig. S1. Definition of the 3-level model parameters: 1 - ground state, 2 - excited state, 3- shelving state, $p_{i}$ denotes the population of the *i*-th state, and $k_{ij}$ is the transition rate from *i*-th to the *j*-th state.

This function is convolved with a Gaussian instrument response function (IRF) to account for timing uncertainty. In our setup, the IRF is dominated by the detector timing jitter 350 ps, resulting in a combined cross-correlation jitter of approximately ~500 ps between the two detection channels. Following convolution, the model is discretized into 0.5 ns bins to match the experimental histogram resolution. Least-squares fits to the experimental data using this convolved model provide the parameters $a$, $\lambda_{1}$, and $\lambda_{2}$ values listed in Table S1.

We use model parameters $a$, $\lambda_{1}$, and $\lambda_{2}$ to obtain the transition rates $k_{ij}$. In the limit where fluorescent relaxation channel dominates, and shelving/deshelving rates $k_{23}$ and $k_{31}$ are much lower $k_{23}+k_{31}\ll k_{21}$, the relations are [1,2]:

$\lambda_{1}=k_{12}+k_{21}$

$\lambda_{2}=k_{31}+\frac{k_{12}}{k_{12}+k_{21}}k_{23}$ (S2)

$a=\frac{k_{12}}{k_{12}+k_{21}}\cdot\frac{k_{23}}{k_{31}}$

The fluorescent relaxation rates $k_{21}$ was taken from the TRPL lifetimes as an input parameter for the 3-level model. The TRPL decays were well described by a monoexponential fit (Section 4.2). Since the three emitters considered here have similar lifetimes, we use a common value $k_{21}=0.291 \mathrm{ns}^{-1}$ for all three. The lifetimes of the shelving state relaxation pathway $\tau_{23}= 1/{k_{23}}$ and $\tau_{31}= 1/{k_{31}}$ are also listed in Table S1.


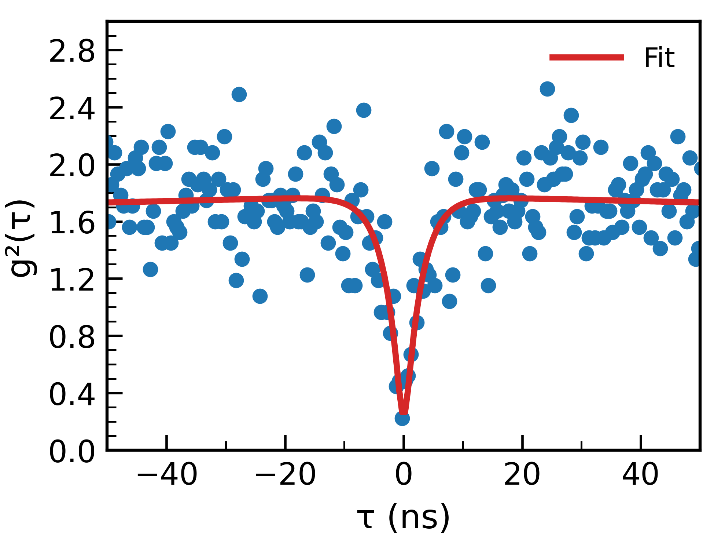

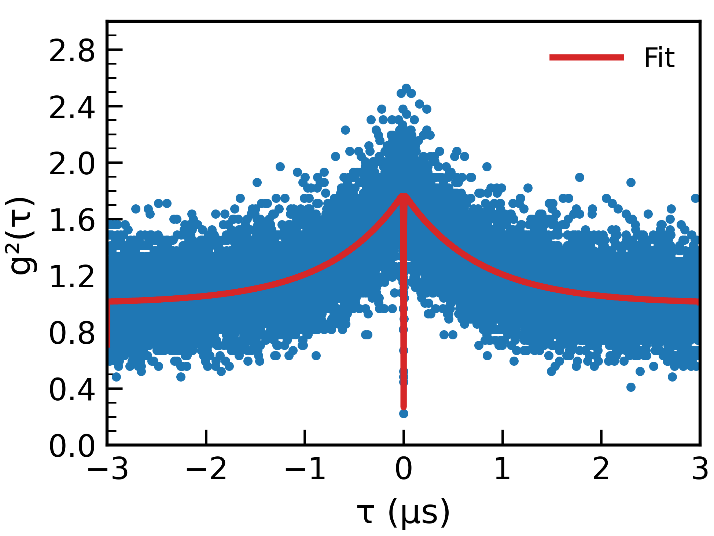


(b)

(a)


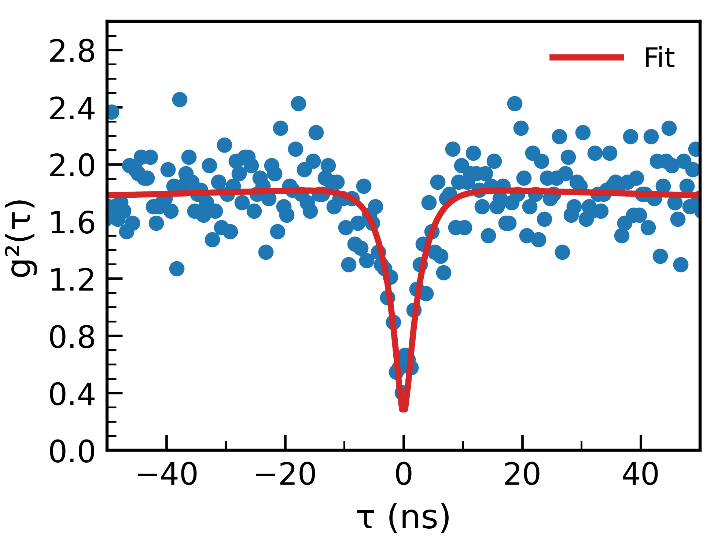

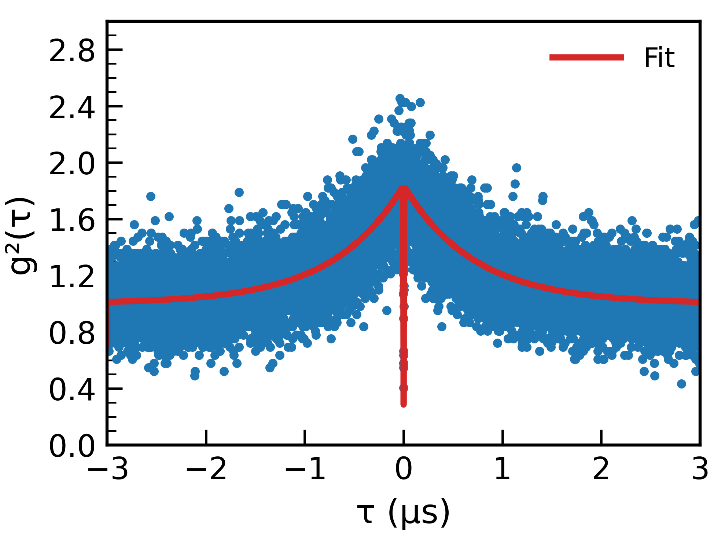


(d)

(c)


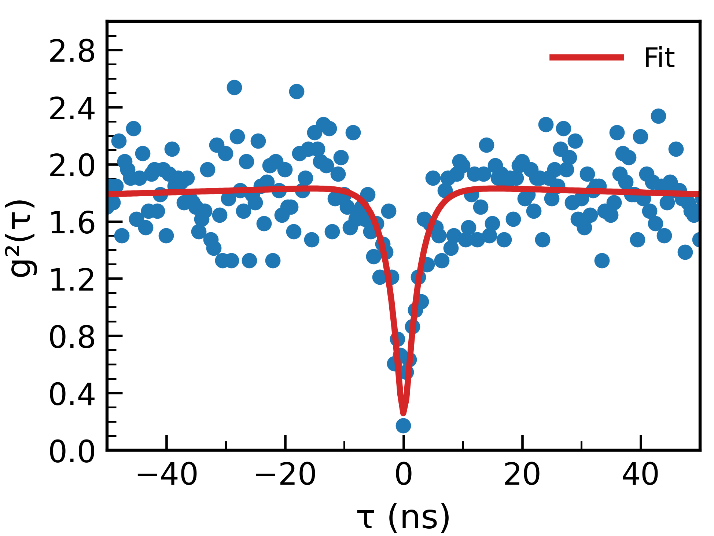

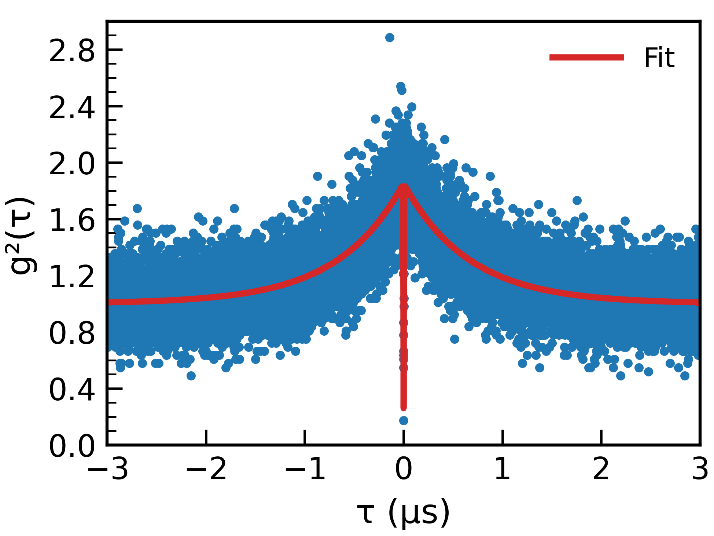


(f)

(e)

Fig. S2. *g*^(2)^ experimental data and 3-level fit of single photon emitters. (a) and (b) correspond to #1 in Table S1. (a) shows antibunching dynamics at shorter time scales and (b) shows bunching at intermediate Stimescales. Similar data for emitter #2 are shown in (c) and (d), and emitter #3: (e) and (f) respectively. Emitter #3 (e-f) is also shown in the main text Fig. 2(f).

Table S1. Results of the g^(2)^ function fit. To calculate of $\tau_{23}$ and $\tau_{31}$ we take the fluorescent transition rate $k_{21}=0.291 \mathrm{ns}^{-1}$.

| **Emitter #** | ***a*** | $\boldsymbol{\lambda}_{\boldsymbol{1}}$**, ns^-1^** | $\boldsymbol{\lambda}_{\boldsymbol{2}}$**, μs^-1^** | $\boldsymbol{\tau}_{\boldsymbol{23}}$**, ns** | $\boldsymbol{\tau}_{\boldsymbol{31}}$**, ns** | ***g*^(2)^(0)** |
| --- | --- | --- | --- | --- | --- | --- |
| 1 | 0.783 ± 0.008 | 0.364 ± 0.027 | 1.33 ± 0.02 | 340 ± 130 | 1340 ± 15 | 0.24 |
| 2 | 0.834 ± 0.007 | 0.379 ± 0.024 | 1.41 ± 0.02 | 360 ± 100 | 1310 ± 12 | 0.26 |
| 3 | 0.853 ± 0.007 | 0.417± 0.028 | 1.52 ± 0.02 | 430 ± 90 | 1220 ± 12 | 0.28 |

**References**

1. Ballesteros, G., Proux, R., Bonato, C., & Gerardot, B. D. readPTU: a Python Library to Analyse Time Tagged Time Resolved Data. (2018). https://github.com/Open-Quantum-Photonics/readPTU.

2. Kitson, S. C., Jonsson, P., Rarity, J. G., & Tapster, P. R. (1998). Intensity fluctuation spectroscopy of small numbers of dye molecules in a microcavity. *Physical Review A*, *58*(1), 620.

3. Fishman, R. E. K. Photon-Emission-Correlation Spectroscopy as an Analytical Tool for Solid-State Quantum Defects. *PRX Quantum* 2023, *4* (1). <https://doi.org/10.1103/PRXQuantum.4.010202>.

1. **Raman data**

Raman measurements were performed to investigate strain and potential changes in the hBN crystal structure after laser irradiation. Measured hBN samples were located on a silica substrate, and the target area was scanned in 0.5 µm steps using 532 nm continuous-wave (CW) excitation, as described in the Experimental Methods section of the main text. The result, averaged over the entire scanned area of one of the samples, is shown in Fig. S3.


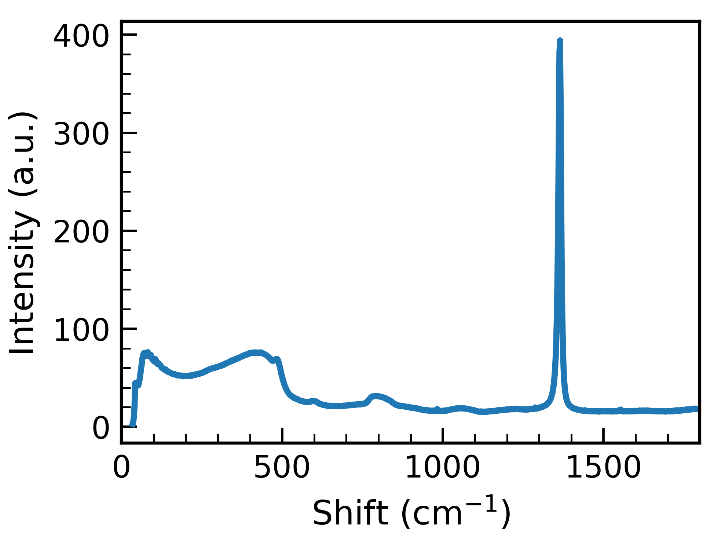

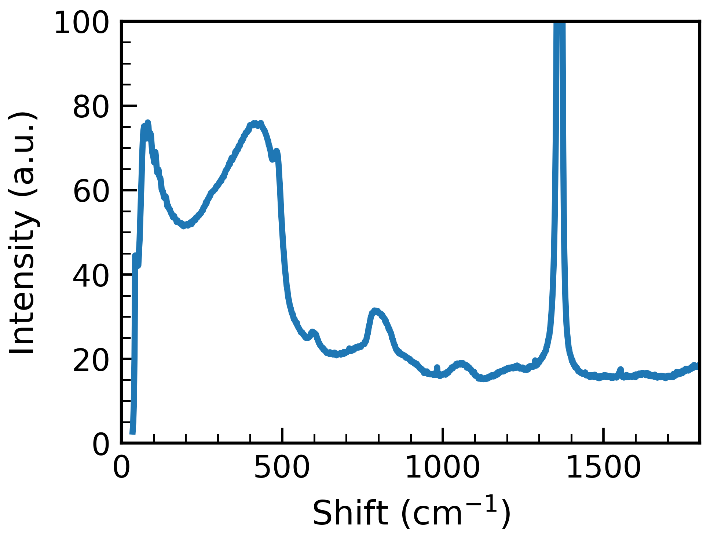


(b)

(a)

Fig S3. Raman spectrum, averaged over the full sample area. (a) full range, (b) zoomed in.

To separate Raman lines from the baseline, we used SNIP (Sensitive Nonlinear Iterative Peak-Clipping) [1] algorithm provided by the pybaselines [2] package. To achieve high-quality background removal in the scan and account for slow background changes, we averaged the spectra in the sliding 3x3 window around the target spectrum.

An example of initial and background-removed data is shown in Fig. S4. In the averaged spectrum the most prominent line is hBN-related E_2g_ at 1367 cm^-1^, associated with in-plane B–N bond stretching, similar to G-band in graphite and graphene. In addition to E_2g_, another Raman-active line in hBN is A_1g_, an out-of-plane vibration, however it is typically a weak low frequency line that was not observed in our experiment. The rest of the Raman structure in Fig. S4 matches reported spectra of silica [3].


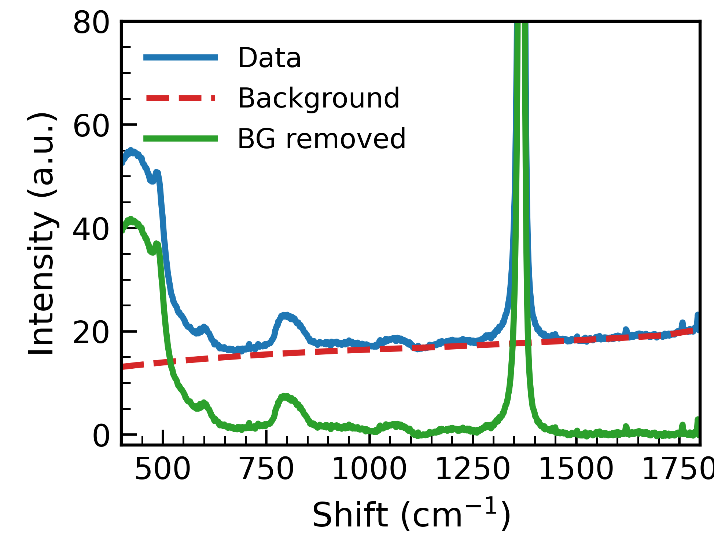


Fig. S4. Illustration of the background removal procedure. The dataset is the same as in Fig. S3.

A few studies have shown traces of a cubic BN phase either after a laser treatment of hBN [4] or after ion implantation [5,6]. Photoluminescence from laser-irradiated sites in cubic BN (cBN) has also been reported [7]. Thus, it is of interest to examine whether cBN phase is present at our laser-irradiated sites on hBN.


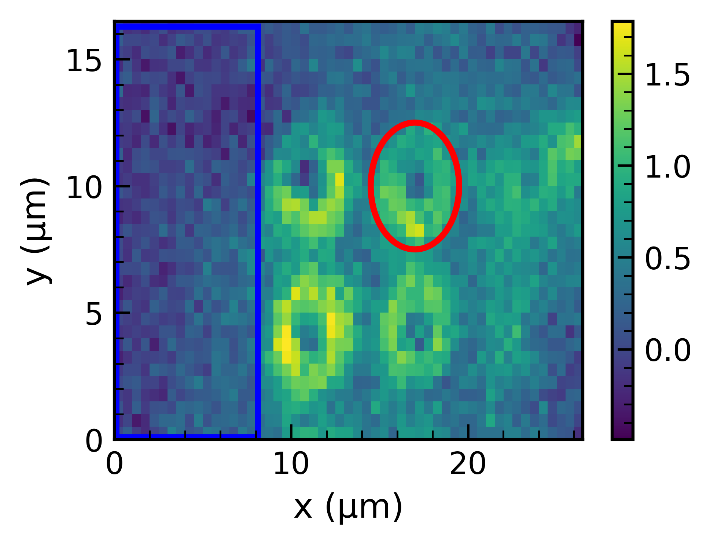

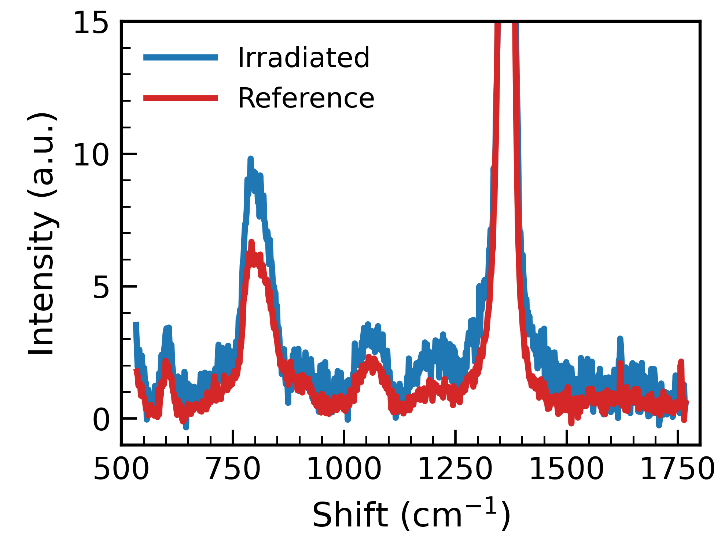


Fig S5. Averaged Raman spectra of the irradiated site (highlighted in red) and reference (highlighted in blue). Irradiated site spectra are chosen within a radius of 3 μm from the center of the irradiated site.

Raman line frequencies associated with cubic BN (cBN) have been reported at 1056 cm^-1^ and 1306 cm^-1^ [8-10]. Theoretical calculations [11] indicate that cBN has a triply degenerate T₂ optical phonon mode at the Γ point, which splits into lower-frequency transverse (TO) modes at 1055 cm⁻¹ and a longitudinal (LO) mode at approximately 1303 cm⁻¹. The higher LO frequency arises from the polar nature of cBN, as LO–TO splitting is typical of polar semiconductors.

We integrated the Raman spectra over the laser-affected areas and compared the results with a non-affected reference. An example of the data from one of the affected sites is shown in Fig. S5. Apart from the change in amplitude, which we attribute to reduced thickness and interference effects in the hBN flake, no evident spectral lines appear near the typical cBN frequencies.

After the background estimate we evaluated the parameters of the main Raman line E_2g_ by fitting it with a single Lorentzian function and extracting its peak position, amplitude, and width. A typical fit result is shown in Fig. S6, indicating that a Lorentzian fit properly represents experimental data.


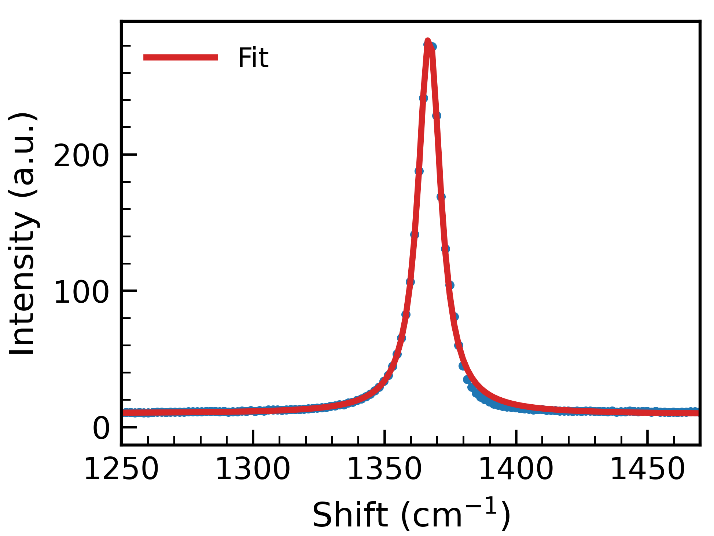


Fig. S6. E_2_*_g_* Raman peak fit with a Lorentzian function.

**References**

1. Morháč, M.; Matoušek, V. Peak Clipping Algorithms for Background Estimation in Spectroscopic Data. *Appl Spectrosc* 2008, *62* (1), 91–106. <https://doi.org/10.1366/000370208783412762>.
2. Erb, D. Pybaselines: A Python Library of Algorithms for the Baseline Correction of Experimental Data, 2025. <https://doi.org/10.5281/zenodo.16790579>.
3. Gerbig, Y. B.; Michaels, C. A. In-Situ Raman Spectroscopic Measurements of the Deformation Region in Indented Glasses. *Journal of Non-Crystalline Solids* 2020, *530*, 119828. <https://doi.org/10.1016/j.jnoncrysol.2019.119828>.
4. Hou, S.; Birowosuto, M. D.; Umar, S.; Anicet, M. A.; Tay, R. Y.; Coquet, P.; Tay, B. K.; Wang, H.; Teo, E. H. T. Localized Emission from Laser-Irradiated Defects in 2D Hexagonal Boron Nitride. *2D Mater.* 2017, *5* (1), 015010. <https://doi.org/10.1088/2053-1583/aa8e61>.
5. Aradi, E.; Naidoo, S. R.; Erasmus, R. M.; Julies, B.; Derry, T. E. Investigations on the Characterization of Ion Implanted Hexagonal Boron Nitride. *Nuclear Instruments and Methods in Physics Research Section B: Beam Interactions with Materials and Atoms* 2013, *307*, 214–217. <https://doi.org/10.1016/j.nimb.2012.12.118>.
6. Aradi, E.; Naidoo, S. R.; Erasmus, R. M.; Julies, B.; Derry, T. E. Raman Studies on the Effect of Multiple-Energy Ion Implantation on Single-Crystal Hexagonal Boron Nitride. *Radiation Effects and Defects in Solids* 2015, *170* (3), 175–182. <https://doi.org/10.1080/10420150.2014.984612>.
7. Buividas, R.; Aharonovich, I.; Seniutinas, G.; Wang, X. W.; Rapp, L.; Rode, A. V.; Taniguchi, T.; Juodkazis, S. Photoluminescence from Voids Created by Femtosecond-Laser Pulses inside Cubic-BN. *Opt. Lett., OL* 2015, *40* (24), 5711–5713. <https://doi.org/10.1364/OL.40.005711>.
8. Lukomskii, A. I.; Shipilo, V. B.; Shishonok, E. M.; Anichenko, N. G. Raman Scattering of Cubic Boron Nitride. 1987, *102* (2), 137–137.
9. Alvarenga, A. D.; Grimsditch, M.; Polian, A. Raman Scattering from Cubic Boron Nitride up to 1600 K. *J. Appl. Phys.* 1992, *72* (5), 1955–1956. <https://doi.org/10.1063/1.351671>.
10. Eremets, M. I. Optical Properties of Cubic Boron Nitride. *Phys. Rev. B* 1995, *52* (12), 8854–8863. <https://doi.org/10.1103/PhysRevB.52.8854>.
11. Ohba, N. First-Principles Study on Structural, Dielectric, and Dynamical Properties for Three BN Polytypes. *Phys. Rev. B* 2001, *63* (11). <https://doi.org/10.1103/PhysRevB.63.115207>.
12. **Polarization-resolved photoluminescence**

Fig. S7 shows additional polarization-resolved PL data of hBN SPEs. Data visualization and analysis follow main text Fig. 6. Two other red emitters are shown here with incomplete polarization suppression, as evident from the spectra at minima-maxima. Furthermore, the PSB-looking feature of SPE in Fig. S7(d-f) may in fact be additional weak emitter, rather than a PSB, as seen from highly offset polarization angle.

`


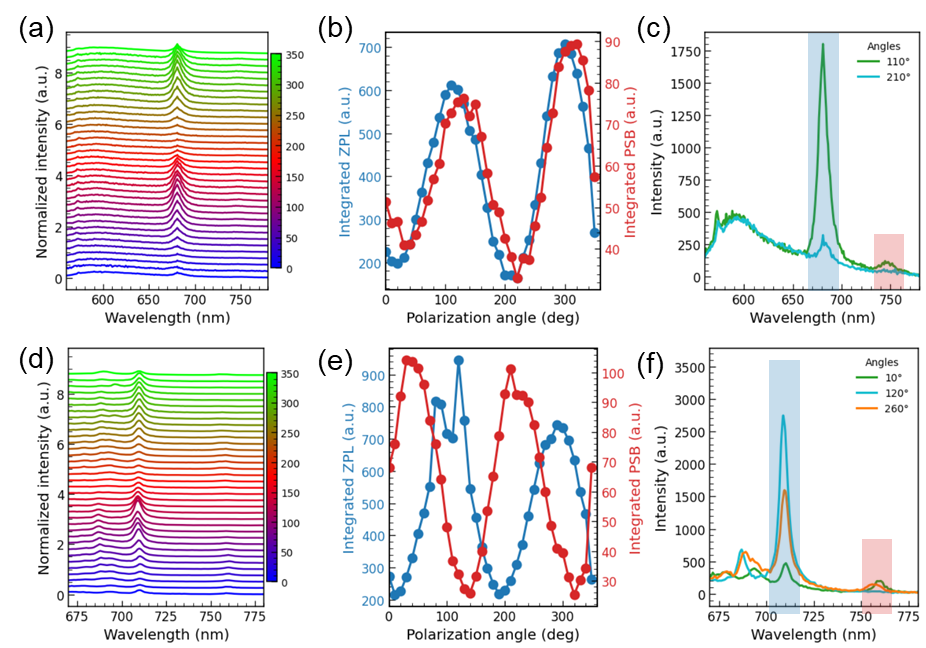


Fig. S7. Polarization-resolved PL data of two SPEs. Data visualization follows main text Fig. 6.
